# Impact of acute kidney injury exposure period among liver transplantation patients

**DOI:** 10.1186/1471-2369-14-43

**Published:** 2013-02-20

**Authors:** Roberto Camargo Narciso, Leonardo Rolim Ferraz, Sergio Mies, Julio Cesar Martins Monte, Oscar Fernando Pavão dos Santos, Miguel Cendoroglo Neto, Cassio José de Oliveira Rodrigues, Marcelo Costa Batista, Marcelino Souza Durão Junior

**Affiliations:** 1 Division of Nephrology, Federal University of São Paulo, São Paulo, Brazil; 2 Liver Unit – Hospital de Transplantes Euryclides de Jesus Zerbini, São Paulo, Brazil; 3 Hospital Israelita Albert Einstein, São Paulo, Brazil; 4 Division of Nephrology – New England Medical Center, Tufts University, Medford, MA, 02155, USA

## Abstract

**Background:**

Acute kidney injury is a common complication of liver transplantation. In this single-centre retrospective observational study, we investigated the impact of acute kidney disease on liver recipient survival.

**Methods:**

The study population consisted of patients who underwent a liver engraftment between January 2002 and November 2006, at a single transplantation centre in São Paulo, Brazil. Acute kidney injury diagnosis and staging were according to the recommendations of the Acute Kidney Injury Network and consisted of scanning the daily serum creatinine levels throughout the hospital stay. Patients requiring renal replacement therapy prior to transplantation, those who developed acute kidney injury before the procedure or those receiving their second liver graft were excluded from the study.

**Results:**

A total of 444 liver transplantations were performed during the study period, and 129 procedures (29%) were excluded. The remaining 315 patients constituted the study population. In 207 procedures, the recipient was male (65%). The mean age of the population was 51 years. Cumulative incidence of acute kidney injury within 48 h, during the first week after transplantation, and throughout the hospital stay was 32, 81 and 93%, respectively. Renal replacement therapy was required within a week after the transplantation in 31 procedures (10%), and another 17 (5%) required replacement therapy after that period. Mean follow-up period was 2.3 years. Time in days from acute kidney injury diagnosis to initiation of replacement therapy or reaching serum creatinine peak was associated with lower overall survival even when adjusted for significant potential confounders (HR 1.03; 95% CI 1.01, 1.05; p=0.002). Overall, patients experiencing acute kidney injury lasting for a week or more before initiation of replacement therapy experienced a threefold increase in risk of death (HR 3.02; 95% CI 2.04, 4.46; p<0.001).

**Conclusions:**

Acute kidney injury after liver transplantation is remarkably frequent and has a substantial impact on patient survival. Delaying the initiation of renal replacement therapy in such population may increase mortality by more than 20% per day.

## Background

Liver transplantation (LT) is the therapy of choice for individuals with advanced chronic liver disease and those with acute liver failure [1]. Patients undergoing LT often suffer from some degree of renal dysfunction [2]. During the perioperative period of LT, they face additional insults to the kidney, such as haemorrhage, administration of nephrotoxic drugs and frequently massive infusion of blood products [3].

Although kidney injury is often reversible, it brings ominous consequences including increased length of hospitalization, time on mechanical ventilation and rate of infection, and progression to chronic renal failure, increasing the total cost of the procedure and directly contributing to a lower liver graft and patient survival [4].

The results of previous studies conducted in transplantation recipients to identify risk factors for developing acute kidney injury (AKI), are not easy to generalize to daily practice given the wide variation in definitions of kidney injury, as well as the periods analysed. The series are small or retrospective, and most of them do not employ the more modern and recommended diagnostic criteria [5].

Several recent studies have suggested that early renal replacement therapy (RRT) improves prognosis in patients who develop AKI [6-9]. Whether these findings are related to the effects of RRT itself, the duration of AKI episode, or the fact that some of the patients started on dialysis earlier would have recovered kidney function and would not require dialysis at all remains debatable.

Considering the high incidence of AKI and the high number of RRT procedures in LT recipients, we proposed this observational study to evaluate the impact of time from AKI onset to RRT initiation or creatinine peak on survival of patients undergoing LT.

## Methods

This study was approved by the Ethics in Research Committee of the Federal University of São Paulo (Protocol number 0358/08). Data were collected from a sequential series of liver transplants performed at the Hospital Israelita Albert Einstein (HIAE) from January 2002 to November 2006 from living and deceased donors. This analysis was limited to this time frame to avoid selection bias, related to the introduction of MELD (Model for End-stage Liver Disease) allocation criteria. Cases taken as urgent, such as patients with acute liver failure or graft primary nonfunction, were prioritized and received the first suitable organ available within the state area [10]. All transplantation recipients were admitted to the intensive care unit (ICU) in the immediate postoperative period. Transplantation organ allocation at the time of the study was ordered chronologically [11].

### Data collection

Based on a prospectively maintained clinical research database, originally developed by the LT surgical team, the medical records for patients undergoing LT from 2002 to 2006 were retrospectively reviewed to retrieve hospitalization data, including baseline demographic characteristics and co-morbid conditions, preoperative clinical and laboratory data, indications of LT, liver disease stage according to the Child-Pugh score, and intraoperative variables including liver graft donor type, surgical time, and total ischaemia period.

A single surgical team from the hepatology group of HIAE, all specifically trained in LT, performed all procedures. The immunosuppressive regimen consisted of a calcineurin inhibitor (cyclosporine predominantly until 2005, later tacrolimus), an antiproliferative drug and a corticosteroid. Whole blood levels of those drugs were measured by fluorescence polarization immunoassay.

Postoperative variables included the development of sepsis, need for pressor drugs, use of well-recognized nephrotoxic agents, including radiocontrast agents, nonsteroidal anti-inflammatory drugs, and antimicrobials (vancomycin, aminoglycosides, polymyxin B and amphotericin B). The Acute Physiology and Chronic Health Evaluation (APACHE) II score was determined at the time of admission to the ICU. MELD score was calculated according to the equation described by Kamath and coworkers [12]. Sepsis was defined according to the international consensus definition [13]. Severe liver graft dysfunction included primary nonfunction and graft dysfunctions with a score higher than 7 in the Gonzales criteria for liver failure [14].

AKI was defined according to the Acute Kidney Injury Network (AKIN) recommendations [15]. Exact dates of AKI event and stage reached in seven days were obtained by computerized scanning of the results of daily laboratory tests for serum creatinine and 24-h urinary output from the electronic medical record system for each procedure on the database, which was divided by four and two, to represent 6-h and 12-h urinary output, respectively. RRT was initiated at the discretion of the nephrology staff, based on common clinical indications such as hypervolemia, hyperkalaemia, refractory acidosis, uremic signs or symptoms, and/or anuria. All attending nephrologists were part of the same team. The procedure report module of the electronic medical record system was used to identify patients who required RRT, and the dates on which they underwent RRT. Patients who developed AKI before LT or required RRT prior to LT were excluded from this analysis, as well as those receiving simultaneous kidney and liver graft or who underwent liver retransplantation.

The main predictor variable of interest for the primary outcome was the time from AKI diagnosis to either initiation of RRT or to creatinine peak for those who did not require RRT. Secondary predictors evaluated were time from each of the AKI stages to RRT or creatinine peak.

The primary outcome in this analysis was patient death after LT from any cause.

### Statistical analysis

Numerical variables were described by median and interquartile range, and categorical variables by absolute and relative frequencies. For comparison of baseline characteristics regarding the main predictor variable, univariable analysis was performed with the chi-squared test and Mann–Whitney U test for categorical and continuous variables, respectively, and the results are shown under p value in Tables 1 and 2.

**Table 1:** Baseline and intraoperative characteristics of the liver transplantation cohort stratified by time from AKI to RRT or serum creatinine peak

	***Time from AKI onset to RRT or SCr peak***			
	***Less than a week (N = 221)***	***A week or more (N = 94)***	***Totals (N=315)***	***p value***
Age (years)	53 (46 – 60)	53 (45 – 58)	53 (46 – 60)	0.502
Weight (kg)	72 (62 – 81)	72 (60 – 82)	72 (62 – 82)	0.538
Height (cm)	169 (162 – 174)	168 (160 – 175)	169 (162 – 174)	0.446
Men	148 (67%)	59 (63%)	207 (66%)	0.472
Diabetes mellitus	42 (19%)	23 (24%)	65 (21%)	0.273
Hypertension	16 (7%)	8 (8%)	24 (8%)	0.697
Smoking habit	10 (4%)	1 (1%)	11 (4%)	0.126
eGFR (MDRD)	92 (74 – 113)	96 (76 – 110)	93 (74 – 112)	0.508
Previous serum creatinine (mg/dl)	0.8 (0.7 – 1.0)	0.8 (0.7 – 1.0)	0.8 (0.7 – 1.0)	0.385
Serum albumin (mg/dl)	3.1 (2.7 – 3.4)	3.1 (2.8 – 3.5)	3.1 (2.7 – 3.5)	0.100
Total bilirubin (mg/dl)	2.6 (1.7 – 4.1)	2.4 (1.6 – 3.8)	2.6 (1.7 – 4.0)	0.419
Main Indication for LT				
Hepatitis B	22 (10%)	12 (13%)	34 (11%)	0.462
Hepatitis C	110 (50%)	48 (51%)	158 (50%)	0.834
Alcoholic cirrhosis	55 (25%)	21 (22%)	76 (24%)	0.629
Acute liver failure	6 (19%)	3 (3%)	9 (3%)	0.816
Liver malignancy	48 (22%)	27 (29%)	75 (24%)	0.182
Other cirrhotic	43 (19%)	18 (19%)	61 (19%)	0.950
MELD score	14 (10 – 18)	12 (9 – 15)	13 (9 – 17)	0.067
Child-Pugh stage				0.075
A	32 (14%)	22 (23%)	54 (17%)	
B	110 (50%)	48 (51%)	158 (50%)	
C	79 (36%)	24 (26%)	103 (33%)	
Deceased liver donor	126 (57%)	55 (58%)	181 (58%)	0.806
Piggyback technique	214 (97%)	92 (98%)	306 (97%)	0.612
Total ischemia time (h)	6.6 (2.4 – 10)	6.9 (2.5 – 10)	6.7 (2.4 – 10)	0.716
Surgical time (h)	7.8 (7 – 8.5)	7.5 (7 – 8.5)	7.6 (7 – 8.5)	0.405
Blood products (units)	4 (2 – 9)	3 (1 – 5)	4 (2 – 8)	0.027
Aprotinin	65 (29%)	18 (19%)	83 (26%)	0.059
Intraoperative pressor	20 (9%)	5 (5%)	25 (8%)	0.262
APACHE II score	15 (13 – 19)	16 (14 – 19)	16 (13 – 19)	0.243

*AKI*: acute kidney injury; *APACHE*: Acute Physiology and Chronic Disease Health Evaluation; *eGFR*: estimated glomerular filtration rate; *LT*: liver transplantation; *MDRD*: Modification of Diet in Renal Disease; *MELD*: Model for End Stage Liver Disease; *RRT*: renal replacement therapy; *SCr*: serum creatinine.

**Table 2:** Postoperative characteristics and outcomes of the liver transplantation cohort stratified by time from AKI to RRT or serum creatinine peak

	***Time from AKI onset to RRT or SCr peak***			
	***Less than a week (N = 221)***	***A week or more (N = 94)***	***Total (N = 315)***	***p value***
Postoperative pressor drugs	41 (19%)	13 (14%)	54 (17%)	0.309
Nephrotoxic drugs	150 (68%)	60 (64%)	210 (67%)	0.486
Sepsis	82 (37%)	38 (40%)	120 (38%)	0.579
Tacrolimus initially	145 (66%)	80 (85%)	225 (71%)	<0.001
Surgical re-intervention	54 (24%)	33 (35%)	87 (28%)	0.053
Severe graft failure	52 (24%)	26 (28%)	78 (25%)	0.437
AKI stage reached				0.005
No AKI	22 (10%)	0 (0%)	22 (7%)	
1	109 (55%)	42 (45%)	151 (48%)	
2	59 (30%)	36 (38%)	95 (30%)	
3	31 (16%)	16 (17%)	47 (15%)	
Required RRT	35 (16%)	13 (14%)	48 (15%)	0.650
Received CRRT	17 (8%)	9 (10%)	26 (8%)	0.579
In-hospital mortality	12 (5%)	8 (8%)	20 (6%)	0.305
Overall mortality	40 (18%)	34 (36%)	74 (24%)	0.001

*AKI*: acute kidney injury; *CRRT*: continuous renal replacement therapy; *LT*: liver transplantation; *RRT*: renal replacement therapy; *SCr*: serum creatinine.

Univariable analysis was performed to identify additional variables associated with primary outcome as potential confounders, with each variable in the database entered into a Cox Proportional Hazards Model as a single covariate with the time to patient’s death as dependent variable. A log minus log plot for each variable was used to check the proportional hazards assumption. Those variables with a p value <0.1 in univariable analysis were subsequently entered into a multivariable model. Time in days from AKI diagnosis and time from each AKI stage to RRT or creatinine peak were entered into a final multivariable model with other confounder variables associated with primary outcome as covariates.

A receiver operator characteristic (ROC) curve relating time from AKI to RRT or creatinine peak with mortality was obtained to identify the optimal cut-off value for conversion of continuous variables into categorical ones, and patients were grouped according the cut-off points of days analyzed by ROC curve. This categorical variable was adjusted for the same factors as the previous model. The same model was applied to a subset of patients who required RRT after LT.

All tests were performed using the statistical software SPSS version 17.0 (SPSS, Chicago, IL).

## Results

### Population demographics and outcomes

During the study period, 444 procedures were performed. Of those, 106 had met AKI criteria before LT; in 16 LT, the recipient had already received a previous liver graft, 5 procedures were simultaneous kidney/liver transplants and in 2 other LT, the patients were on the chronic RRT program prior to LT. These 129 LT (29%) were excluded from analysis. The remaining 315 LT were performed in 315 patients, who represented the study population. Baseline and postoperative characteristics are listed in Tables 1 and 2.

Severe AKI, requiring RRT occurred after 48 procedures (15%), of which 31 (10%) were within the first week after transplantation with a median of 3 (3–7) days. Another 17 (5%) required RRT after that period with a median of 15 (10–21) days. Continuous RRT (CRRT) was the initial therapy for 26 (8.3%) procedures, CRRT only in 11 of them (3.5%), and followed by intermittent haemodialysis in 15 (4.8%). Intermittent haemodialysis alone was the therapy of choice for 22 (7%) patients. All patients requiring RRT fulfilled criteria for the AKI before the initiation of therapy. Mortality rate for those requiring RRT after LT was 58% (n=28), while for those not requiring it was only 17% (n=46) (OR 6.7; 95% CI 3.49, 12.96; p<0.001).

Mortality rates observed in the hospital, up to 90 days and up to 1 year after LT were 6.3% (n=20), 10% (n=32) and 18% (n=56), respectively. The overall mortality rate was 24% (n=74) after a mean period of 1488 days or 4.1 years. All cases alive at the end of the follow-up were censured on April 1st, 2007.

### Mortality risk factors

Time from diagnosis of AKI to RRT or creatinine peak was significantly associated with the primary endpoint (p<0.05). In multivariable analysis, AKI to RRT or creatinine peak (hazard ratio [HR] 1.03; 95% CI 1.01, 1.05; p=0.003), remained significant even after adjustment for other risk factors associated with the primary outcome.

By generating a ROC curve with mortality as the dependent variable, the optimal cut-off value for days from AKI to RRT or creatinine peak was determined to be seven days after AKI onset. The resulting categorical variable with this cut-off discriminated mortality with a sensitivity and specificity of 44% and 77%, respectively, giving an area under the curve of 0.607.

Baseline and postoperative variables grouped by the interval between AKI diagnosis to RRT initiation or creatinine peak greater or equal to or less than 7 days for cohort subset analysed with respective p values are shown in Tables 1 and 2.

**Figure 1:**
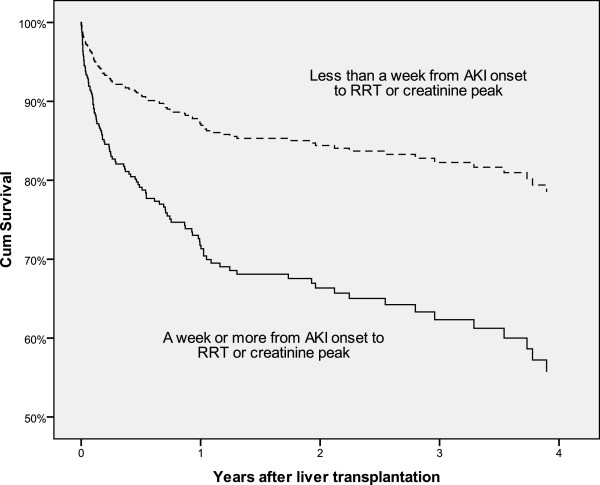
**Cox regression survival curve.** Adjusted survival curve for whole cohort grouped by days to RRT or creatinine peak; p<0.001.

The adjusted categorical predictor for main confounders remained strongly associated with higher risk of death (HR 3.02; 95% CI 2.04, 4.46; p<0.001). This association remained unchanged even after adjustment for each clinically relevant or statistically significant confounder for all procedures in this cohort (Figure 1), as well as when analysing only those patients requiring RRT (Table 3). This group of patients experienced a nearly threefold increase in risk of death even when adjusted for every confounder.

**Table 3:** Associations of predictors with primary outcome (mortality)

***Predictor***	***HR***	***95% CI***	***P-value***
A week or more from diagnosis of AKI to RRT or SCr peak			
Unadjusted	3.11	1.92, 5.05	<0.001
Adjusted for age, gender, diabetes, hypertension, smoking habit and liver malignancy	3.03	1.84, 4.98	<0.001
Adjusted for age, gender, diabetes, hypertension, smoking habit, liver malignancy, total surgical time, postoperative pressor drugs and severe liver graft failure	3.68	2.16, 6.26	<0.001
A week or more from AKI to RRT only, adjusted^1^	2.93	1.17, 7.30	0.021
Days from AKI diagnosis to RRT or creatinine peak adjusted	1.03	1.01, 1.05	0.002
Days from AKI stage 1 to RRT or creatinine peak adjusted	1.03	1.01, 1.05	0.002
Days from AKI stage 2 to RRT or creatinine peak adjusted	1.19	1.10, 1.28	<0.001
Days from AKI stage 3 to RRT or creatinine peak adjusted	1.18	1.08, 1.30	0.001

*AKI*: acute kidney injury; *CI*: confidence interval; *HR*: hazard ratio; *RRT*: renal replacement therapy; *SCr*: serum creatinine; ^1^ model applied only to patients who required RRT after LT.

Moreover, using continuous time from the beginning of each AKI stage to RRT or creatinine peak, as secondary predictors, while adjusting for the same confounders, we found a successively higher HR. HR transformations revealing the time that doubles the risk of death is shown in Table 4.

**Table 4:** Time to double risk of death according to AKI stage reached

***AKI stage***	***HR***	***Time in days to double the risk of death***	***95% CI***
Stage 1	1.03	23	14 – 70
Stage 2	1.19	4	3 – 7
Stage 3	1.18	4	3 – 9

*AKI*: acute kidney injury; *CI*: confidence interval; *HR*: hazard ratio.

## Discussion

AKI is well recognized for its impact on the evolution of critically ill patients. In the various severity scores such as the APACHE II and Sequential Organ Failure Assessment score, AKI alone is responsible for 15-20% of the total score weight [16]. The diversity of definitions is responsible for large discrepancies in AKI incidence and mortality [17]. Using the AKIN criteria, we observed a high incidence of renal dysfunction within the first 48 h after LT, requiring RRT in 20% of the cases. Most RRT were performed within the first week after transplantation. The incidence of AKI after LT described in the literature is quite wide, with reports ranging from 8.3 to 61% [4,18]. Lafayette described 27 cases of AKI (23%) in 115 liver transplants, which were associated with longer stay in the ICU, higher risk of infection, and higher cost and in-hospital mortality (46% in group AKI vs. 9% of patients without AKI) [19]. The overall rate observed in this series was higher than in other similar works, due to criteria used to define AKI. In-hospital mortality was substantially lower than that reported in that study for both groups, which suggests that there may have been technical improvements in hospital care after the publication of that study.

In the general population developing AKI in the absence of hyperkalaemia or hypervolemia, the threshold for blood urea nitrogen (BUN) or creatinine for RRT initiation remains subjective, and no additional benefit is associated with earlier initiation of therapy [20]. It has also been argued that earlier initiation of RRT could mean exposure to the risks of therapy in patients who would regain renal function with conservative therapy alone Also, intradialytic hypotension episodes may delay recovery of renal function and adversely impact patient survival [21]. Several studies over the last decade have evaluated the impact of time of initiation of RRT on the outcome of AKI in the general population. Gettings and colleagues analysed the time of initiation of CRRT on the outcome in patients with posttraumatic AKI and stratified 100 consecutive patients based on BUN at baseline [22]. Survival was 39% in the “early” initiation group compared to 20% in the “late” group. Nevertheless, RRT has been frequently reported as an independent predictor of mortality [23]. These findings have been often evoked to delay RRT initiation, as if RRT itself were responsible for higher death rate. In fact, according to these findings, each day of delaying RRT initiation increases the risk of death from 3% to 19%.

In studying 1238 patients, distributed in three different groups: early (<2 days), delayed (2–5 days) or late (>5 days) RRT initiation after ICU admission, Bagshaw and coworkers observed greater mortality in the late group. The same held true when comparing the creatinine levels on RRT initiation, that is, no assumptions regarding time of exposure to AKI could be made [7]. The study of Andrade and coworkers also supports early intervention as being beneficial in a population of patients developing AKI after leptospirosis infection, comparing intensive and promptly initiated therapy versus less intensive and delayed therapy, intensive group performed better, but the study did not take into account the exposure period to severe stages of AKI [24]. Payen and colleagues evaluated the effects of excessive positive fluid balance on 60-day mortality, the mention of early vs. late RRT initiation refers to time in days since ICU admission, and again, no assumptions could be made regarding AKI exposure period [25]. Ostermann compared patients initiating RRT before reaching AKI stage 3 with patients who did not reach that stage prior to RRT initiation. Their cut-offs were set for the period before RRT after ICU admission, regardless of AKI stage, or time from AKI stage 3, no matter how long the patient had been exposed to milder AKI stages.

Coca and colleagues retrospectively studied the duration of AKI episodes in more than 35,000 diabetic patients undergoing noncardiac surgery in multiple US centres and found higher durations associated with increased risk of long-term mortality [26]. According to these authors, an AKI episode occurred when the patient met at least AKI stage 1 criteria or was on RRT, leading to overestimation of AKI episode duration, and RRT exposure was equivalent to AKI exposure, which may not hold true in most situations.

One could argue this analysis should be limited to patients who need RRT [27]. When analysing the RRT subgroup only, the association of mortality with longer time of AKI onset to therapy was still significant, but the theoretical main predictor was, in fact, the exposure period to AKI, and we considered the exposure period of AKI onset to RRT initiation to be the same as AKI onset to creatinine peak. Although a crude marker of kidney function, the creatinine peak was used to indicate the beginning of kidney function recovery, and consequent reduction of the effects of AKI exposure, similarly to RRT initiation. This approach is backed up by literature findings implicating small rises in creatinine to the increase in mortality [28]. At least one study found that mortality rates are similar whether the patient has an increase or a decrease in serum creatinine levels of 0.3 mg/dl or more during the study period [29]. Although the published AKIN criteria specify an absolute increase of ≥0.3 mg/dL within 48 h, the authors applied the criteria without specifying the direction of change, as they classified patients with falling creatinine values as having AKI as well. The authors considered that to be related to the fact that few, if any, interventions are able to improve renal function and consequently lower serum creatinine levels, if it is chronically reduced, and therefore, any improvement found over short periods of time could be related to a previous episode of AKI [29]. Even though the validity of such equivalence (RRT initiation vs. kidney function recovery) is debatable regarding the effect magnitude, one must consider the much lower side effects of kidney function recovery when compared to RRT side effects.

Literature evidence suggests that there is excess mortality independently attributed to AKI itself, and one of the possible explanations for that is the effect of uremic toxins on the patient’s homeostasis and inflammatory response [30,31]. We proposed that longer periods of exposure to AKI would increase their effects and mortality. Considering that, RRT was used only to censure such period, as serum creatinine measurements after RRT initiation would no longer reflect the patient’s glomerular filtration rate and/or severity of kidney injury.

As is the case with past observational studies on this topic, this study was not able to address the issue of whether the association of earlier RRT initiation with improved outcomes is related to the fact that some of the patients started on dialysis earlier would have recovered kidney function and would not have required dialysis at all, since the inclusion of such patients in the “early” initiation group may result in improved outcomes. We minimized that issue by including patients who recovered kidney function without RRT. Hardly any evidence in this field has come from non-observational studies. Even in the only available randomized controlled trial, no strong conclusion could be drawn, since early versus late was defined by the urinary output versus biochemical values [32]. While the real impact of early versus late initiation of dialysis would only be justified when, at the moment patients fulfilled the criteria for dialysis, one group started RRT while the other group was kept under clinical treatment for a longer period. Even so, other confounders may arise, as an intention-to-treat approach must be employed to avoid the ethical dilemma of not treating potentially fatal conditions in the group randomized for clinical treatment.

Furthermore, our study design was not able to control for other unrecorded confounders, but when we applied a propensity score for every possible confounder we had in our database. The resultant Cox model for mortality, with the propensity score for a longer AKI exposure, demonstrated a week of exposure to AKI effects had an even higher HR (data not shown).

In spite of these limitations but considering that most of the previous observational studies lacked common criteria to identify AKI, that they did not employ modern criteria to define AKI within a narrow timeframe as recommended by AKIN group, and they were usually limited to an AKI population requiring RRT, most of them too critically ill to experience any benefit from any therapy, we took a different approach. Instead of studying early versus late RRT initiation time, we decided to study the effect of time of exposure to AKI episodes throughout the whole hospital stay, regardless of the need for RRT, which was initiated on the basis of clinical criteria. We demonstrated that regardless of AKI stage reached, patients developing AKI lasting 7 days or longer, before beginning of recovery or initiation of RRT, have a threefold risk of dying, reflecting the impact of exposure period to kidney injury on patient’s outcome. By applying the inverse transformation to obtain the HR from this categorical predictor, we found that each day of delay in RRT initiation increases the risk of death by 20%, with a twofold increase in risk of dying occurring after 4 days of AKI onset.

## Conclusions

In conclusion, our findings suggest a higher mortality in LT recipients with longer exposure period to AKI, especially for those reaching AKI stage 2 or 3 with AKI episodes lasting longer than four days. In such group, lack of recovery of kidney function or initiation of RRT seems to double the risk of death. Whether these findings translate well into clinical practice requires additional evaluation such as a randomized controlled trial. Delaying RRT initiation seems to be unjustifiable, especially in this population where absolute RRT indications may take longer to occur.

## Consent

Approval was obtained from local Ethics Committee, and formal informed consent was waived because of the observational nature of the study.

## Abbreviations

AKI: Acute kidney injury; AKIN: Acute Kidney Injury Network; APACHE: Acute Physiology and Chronic Health Evaluation; BUN: Blood urea nitrogen; CI: Confidence interval; CRRT: Continuous renal replacement therapy; HIAE: Hospital Israelita Albert Einstein; HR: Hazard ratio; ICU: Intensive care unit; LT: Liver transplantation; MELD: Model for End-stage Liver Disease; OR: Odds ratio; ROC: Receiver operator characteristic; RRT: Renal replacement therapy.

## Competing interests

The authors declare that they have no competing interests.

## Authors’ contributions

LRF and SM provided the original database. RCN participated in the design of the study, performed the statistical analysis and drafted the manuscript. MSD conceived the study and MCB participated in the study design and coordination and helped to draft the manuscript. JCM, OFPS, MCN and CJOR reviewed the manuscript draft. All authors read and approved the final manuscript.

## Pre-publication history

The pre-publication history for this paper can be accessed here:

http://www.biomedcentral.com/1471-2369/14/43/prepub
